# Glucagon-Induced Acetylation of Energy-Sensing Factors in Control of Hepatic Metabolism

**DOI:** 10.3390/ijms20081885

**Published:** 2019-04-16

**Authors:** Li Zhang, Weilei Yao, Jun Xia, Tongxin Wang, Feiruo Huang

**Affiliations:** Department of Animal Nutrition and Feed Science, College of Animal Science and Technology, Huazhong Agricultural University, Wuhan 430070, China; zhangli302@webmail.hzau.edu.cn (L.Z.); yaoweilei0728@gmail.com (W.Y.); xiajunkris@gmail.com (J.X.); michaelwangtongxin@gmail.com (T.W.)

**Keywords:** glucagon, acetylation, deacetylation, energy-sensing factors, hepatic metabolism

## Abstract

The liver is the central organ of glycolipid metabolism, which regulates the metabolism of lipids and glucose to maintain energy homeostasis upon alterations of physiological conditions. Researchers formerly focused on the phosphorylation of glucagon in controlling liver metabolism. Noteworthily, emerging evidence has shown glucagon could additionally induce acetylation to control hepatic metabolism in response to different physiological states. Through inducing acetylation of complex metabolic networks, glucagon interacts extensively with various energy-sensing factors in shifting from glucose metabolism to lipid metabolism during prolonged fasting. In addition, glucagon-induced acetylation of different energy-sensing factors is involved in the advancement of nonalcoholic fatty liver disease (NAFLD) to liver cancer. Here, we summarize the latest findings on glucagon to control hepatic metabolism by inducing acetylation of energy-sensing factors. Finally, we summarize and discuss the potential impact of glucagon on the treatment of liver diseases.

## 1. Introduction

Health problems associated with obesity, type 2 diabetes (T2DM), and nonalcoholic fatty liver disease (NAFLD) are becoming more arresting by the day [1,2,3]. Abnormal physiological control could destroy metabolic balance and would eventually give rise to these chronic acquired diseases [4,5]. Overnutrition or malnutrition have been revealed to be linked to chronic inflammation and may result in disorders of energy balance in the body [6,7]. As the hub of metabolism, the liver has an abundant blood supply, and its unique morphological structure makes its metabolism extremely active [8]. It is also closely related to various tissues and organs in the metabolism of glucose, lipids, protein, vitamins, and hormones, etc. Consequently, the liver is responsible for maintaining somatic harmony. It does so by precisely controlling the metabolism of glucose and lipids [9]. Hepatic metabolic disorders are strongly linked to the existence of liver diseases. Perpetual obesity and overnutrition inflict inflammation in the liver and invite many metabolic disorders and diseases. These effects eventually contribute to the appearance and metastasis of liver cancer, which is currently the leading cause of death from liver disease [10,11].

A growing body of studies have uncovered that some transcription factors play significant roles in controlling liver energy metabolism. After undergoing post-translational modifications (PTMs), the activity and stability of these transcription factors will be altered, thereby affecting their biological functions in the liver [12]. During the past decades, many researchers have indicated that acetylation had become a critical post-translational modification in cell regulation, particularly by modifying histones and nuclear transcriptional factors. Lysine acetylation is an evolutionarily, highly conserved post-translational modification mechanism. Histone acetylation under the control of lysine acetyltransferases (KATs) and histone deacetylases (HDACs), and the dynamic balance of these two activities, is the linchpin in maintaining homeostasis [13,14]. 

Recent mass spectrometry has unmasked that almost all metabolism enzymes are acetylated, indicating that acetylation has a broad regulatory effect on cellular metabolism. Furthermore, acetylation also participates in various biological processes, such as energy metabolism, signal transduction, and oxidative stress, by altering the protein–protein interactions, protein stability, catalytic activity, and subcellular localization of metabolic enzymes [15,16]. Regulation of metabolic pathways by acetylation is important for the occurrence and development of metabolic-related diseases such as obesity, cardiovascular disease, diabetes, and tumorigenesis [17,18]. Predictably, as a vital metabolic organ, most metabolic processes in the liver are subjected to acetylation, such as glycolipid metabolism and urea cycles. The acetyltransferase and deacetylase enzymes are affected by the by nutritional levels, so they can quickly respond to the liver energy balance.

Metabolism of nutrients in the liver is under the control of glucagon and insulin. This is the reason the interaction between insulin and glucagon is able to maintain the body’s energy balance [19]. Glucagon is a polypeptide synthesized and secreted by pancreatic alpha cells. The primary physiological role of glucagon is to fight insulin and induce hepatic glucose production, thereby maintaining glucose balance in the liver. Previous research has demonstrated that most liver metabolic diseases are concomitant with an increase in plasma glucagon concentration [20,21,22,23]. Recently, some researchers have shown that glucagon regulates liver metabolism by controlling acetylation of energy-sensing factors. Energy-sensing factors are capable of making the corresponding transformation according to different energy levels of the body to maintain energy balance [24]. For example, in the fasting state, glucagon regulates the expression of forkhead box o1 (FOXO1) and cyclic AMP (cAMP)-response element binding protein (CREB)-regulated transcription coactivator 2 (CRTC2) by inducing acetylation, thereby increasing the expression of gluconeogenesis-related genes to regulate glucose metabolism [25,26,27,28]. Glucagon additionally induces the acetylation of sterol regulatory element binding protein-1c (SREBP-1c) and cAMP-responsive element binding protein H (CREBH) to regulate lipid metabolism [29,30,31,32]. Additionally, in the pathological state of the liver, glucagon-induced acetylation of energy-sensing factors such as signal transducer and activator of transcription–3 (STAT3) provide a potential treatment strategy for liver disease [33] (Table 1). This review primarily focuses on how glucagon controls hepatic metabolism by altering the acetylation status of energy-sensing factors.

## 2. Glucagon-Mediated Glucose Homeostasis in the Liver

**Figure 1:**
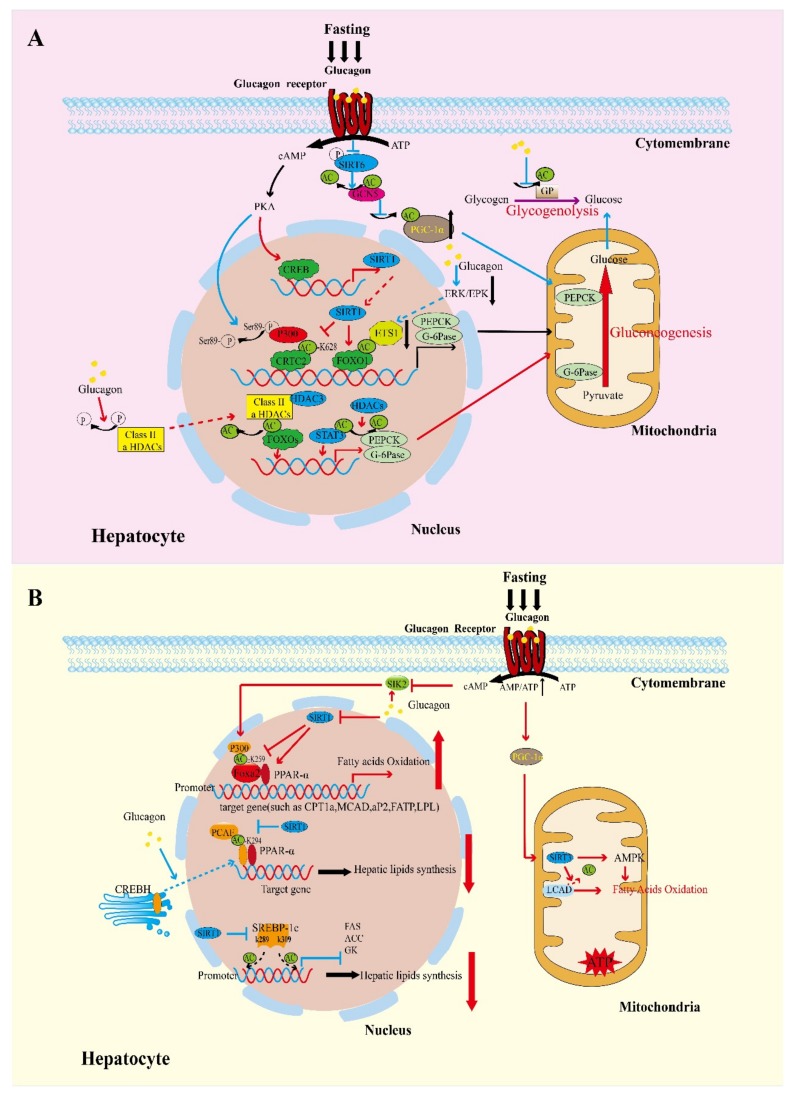
Glucagon-induced acetylation of energy-sensing factors in control of hepatic glycolipid metabolism. (**a**) Blue arrow: Glucagon initiates the transcription of downstream G6Pase and PEPCK1 by inducing acetylation of CRTC2 and FOXO1 and reducing acetylation of PGC-1α and GP, which leads to elevating gluconeogenesis. Red arrow: Glucagon-induced deacetylation of CRTC2 and FOXO have different roles in glucose metabolism. (**b**) Blue arrow: Glucagon-induced acetylation of CREBH and SREBP-c1 inhibit the hepatic lipids synthesis. Red arrow: Glucagon-induced acetylation of PPAR-α and Foxa2 increase fatty acid oxidation. (G6Pase: Glucose-6-phosphatase; CRTC2: CREB regulated transcription coactivator 2; FOXO1: Forkhead box O1; PEPCK1: Phosphoenolpyruvate carboxykinase; GP: Glycogen phosphorylase; PGC-1α: Peroxisome proliferator-activated receptor gamma coactivator 1α; PPAR-α: Peroxisome proliferator-activated receptor-α; SREBP-1c: Sterol regulatory-element-binding protein-1c; CREBH: cAMP-responsive element-binding protein H).

As an essential nutrient of the body, glucose is the primary source of energy for many cells and is dependent on blood for transportation. Therefore, maintaining blood glucose balance is significant for ensuring the nutritional supply and normal metabolic activities of various tissues and organs. The liver is the main organ that critically maintains blood glucose balance. It takes up glucose through glycogen production and releases glucose through gluconeogenesis [64]. During fasting or caloric restriction (CR), the liver maintains energy supply by enhancing glycogenolysis and gluconeogenesis [65]. Meanwhile, glucagon stimulates transcription of the gluconeogenesis gene through various ways in the fasting state, one of which is acetylation of energy-sensing factors (Figure 1A).

### 2.1. Glucagon-Induced Acetylation Regulates Hepatic Gluconeogenesis during the Fasting State

During fasting and CR, the liver provides glucose to tissues and organs through glycogenolysis and gluconeogenesis to ensure healthy metabolism of the body [66]. The gluconeogenesis process is regulated by nutrient levels and various hormones [67]. Several transcription factors and coactivators are engaged in this process after being acetylated by glucagon induction. Glucagon promotes dephosphorylation of the Ser89 site of p300 via the cAMP-dependent protein kinase (PKA) pathway, thereby increasing p300 activity [25]. p300 has histone acetyltransferase activity, where it transfers an acetyl group to the lysine residue, which enhances the activity of CRTC2 by acetylating the Lys628 site of CRTC2 [25,68,69]. As the switch protein for blood glucose regulation in humans, CRTC2 is sensitive to hormones and glucose levels, mainly expressed in the liver and kidney [70]. Therefore, glucagon initiates the transcription of downstream glucose-6-phosphatase (G6Pase) and phosphoenolpyruvate carboxykinase (PEPCK1) by acetylating CRTC2 via p300, such that it enhances gluconeogenesis to maintain energy balance [25,34,71]. Additionally, Anne and colleagues showed that mRNA and protein levels of FOXO1 were elevated prominently in mice livers after fasting [25]. FOXO1 can also promote transcription of gluconeogenic enzyme genes such as G6Pase and PEPCK1, which in turn leads to elevating gluconeogenesis [28,68]. Remarkably, silence of coactivator p300 leads to a decrease in mRNA and protein levels of FOXO1. In addition, suppression of histone acetyltransferase activity of p300 prominently reduces mRNA and protein levels of FOXO1 in the liver of fasting mice and fasting blood glucose levels [26,35]. Accordingly, we conclude that glucagon might elevate the FOXO1 gene and CRTC2 expression in the fasting state via p300, and the expression of FOXO1 and CRTC2 would further increase gluconeogenesis. A recent study also revealed that Ets1-mediated acetylation of FOXO1 responds to glucagon signaling to regulate gluconeogenesis in the fasting state [36]. During fasting, glucagon down-regulates the activity of Ets1 via the mitogen-activated protein kinase kinase (MEK) extracellular signal-regulated kinase (ERK) pathway [36,72]. FOXO1 is acetylated by Ets1 and leads to its incapacity of binding to gluconeogenic promoters. Therefore, glucagon inhibits the process of Ets1 acetylation of FOXO1 to increase gluconeogenesis.

A recent finding illustrates that glucagon plays a significant role in control of the process where general control nonrepressed protein 5 (GCN5) acetylates peroxisome proliferator-activated receptor gamma coactivator 1-alpha (PGC-1α) in the fasting state [73,74]. As one regulator of gluconeogenesis, PGC-1α effectively stimulates hepatic gluconeogenesis by increasing the expression of gluconeogenic genes such as PEPCK and G6Pase [37]. GCN5 can acetylate PGC-1α and decrease its activity, such that PGC-1α cannot bind to the promoter of its target gene, which leads to decrease of gluconeogenesis [74]. Surprisingly, as one of the deacetylases, sirtuin 6 (SIRT6) increases PGC-1α acetylation and downgrades hepatic glucose production (HGP). The reason is that SIRT6 increases the degree of acetylation of PGC-1α through the deacetylation and activation of GCN5 [38]. In the fasting state, glucagon down-regulates the expression of SIRT6 by phosphorylation, resulting in inhibition of GCN5 activity [38,39]. Glucagon reduces the degree of acetylation of PGC1-α by restraining GCN5, thereby transmitting the signal of cellular energy status to PGC-1α, accordingly increasing the output of cellular energy by increasing the expression of gluconeogenesis genes. In summary, this finding reveals an interesting phenomenon in which glucagon is able to participate in the acetylation process of the energy-sensing factors PGC-1α by regulating the SIRT6.

Additionally, glucagon lessens glycogen phosphorylase acetylation to promote hepatic glycogen phosphorylase (GP) activity. GP acts as a catalytic rate-limiting enzyme in the glycogenolysis process and plays an essential role in preserving glucose homeostasis [75,76]. Zhang et al. unmasked that the activity of GP was reduced after being modified by acetylation. Afterward, they recorded the acetylation levels of GP expressed in hepatocytes after treatment with glucagon, and the results revealed that GP acetylation was decreased by glucagon induction. Finally, their research unmasked that glucagon induced the decreasing acetylation of GP and led to higher GP activity, resulting in increased production of glucose by glycogenolysis [40]. However, it is unclear which enzymes are involved in this process, and the precise mechanism by which glucagon regulates GP acetylation remains to be elucidated. 

### 2.2. Glucagon-Induced Deacetylation Regulates Hepatic Gluconeogenesis during the Fasting State

Glucagon also regulates glucose balance by inducing deacetylation of energy-sense factors. Sirtuins are a class of NAD^+^-dependent deacetylases, and their functions are closely related to cellular metabolism [77]. There are seven recognized members of the human sirtuin family, which can interact with p53, FOXO1/PGC-1α, Nuclear factor kappa B (NF-κB), and other proteins to regulate cellular stress response, metabolism, aging, and apoptosis [30]. In the past few decades, research on biological functions of the sirtuin family has made considerable progress. In this progress, sirtuin 1 (SIRT1) was studied wildly in many aspects. SIRT1 deacetylates histones and many important transcription factors to act as an energy-sensing factor in hepatic energy metabolism [78,79,80]. Lilia et al. provided in vivo evidence that glucagon increases SIRT1 expression through CREB activation in the fasting state. In response to CR, glucagon promotes energy production through PKA-mediated activation of the CREB, and CREB is capable of binding to the SIRT1 promoter to increase its transcription [81]. 

As mentioned above, glucagon increases the activity of CRTC2 through p300 to increase gluconeogenesis in the fasting state. In fact, short-term fasting can increase CRTC2–p300 interaction in the liver, while long-term fasting destroys it through SIRT1. During prolonged fasting, glucagon impels SIRT1 to deacetylate CRTC2 and promotes CRTC2 ubiquitin-dependent degradation with constitutively photomorphogenic 1 (COP1). Meanwhile, SIRT1-mediated deacetylation increases the activity of FOXO1 to promote expression of the glycogen production program [25]. Additionally, studies have shown that SIRT1 activators reduce gluconeogenesis in insulin-resistant animals. Paradoxically, it also increases the activity of FOXO1, and the increase of FOXO1 will lead to increased gluconeogenesis [82,83,84]. During this time, upregulation of FOXO1 activity by SIRT1 appears to be critical for maintaining energy balance. Interestingly, activation of CRTC2 by glucagon is abolished by deacetylation of CRTC2 by SIRT1 in prolonged fasting, and SIRT1 has a positive regulatory effect on SIRT6, all of which will inhibit the production of glucose [85,86,87]. It seems contradictory that SIRT1 can both promote and inhibit gluconeogenesis. In fact, several studies about the function of SIRT1 in the liver metabolism have found that SIRT1 plays a diametrically opposite role hepatic glucose metabolism during different physiological periods. Also, the notion has been put forward that prolonged stimulation of SIRT1 expression might tone down the gluconeogenic program through deacetylation and inhibition of CRTC2, thereby favoring energy-sparing processes such as ketogenesis [25,84,88,89,90]. This result reflects that glucagon-induced deacetylation through SIRT1 has different regulatory effects on glucose production in different fasting stages.

HDACs, a class of proteases that deacetylate histone, contribute to chromosome structural modification and regulation of gene expression [91]. HDACs regulate the expression of crucial gluconeogenesis enzymes by controlling the activity of the forkhead transcription factor (FOXO) family, thereby regulating gluconeogenesis. For example, HDAC1 induces the expression of hepatocyte nuclear factor 4α (HNF4α), leading to the dephosphorylation of FOXO1 into the nucleus of hepatocytes, which in turn prompts the expression of staple enzymes in the liver and increases glycogenesis. Recent studies have found that class IIa HDACs were also involved in the regulation of FOXO family activity to regulate hepatic gluconeogenesis [41]. In the fasting state, glucagon rapidly dephosphorylates class IIa HDACs and transfers it from the cytoplasm to the nucleus, thereby recruiting HDAC3 to form a complex and promoting deacetylation of FOXOs. This enhances its transcriptional activity and induces transcription of the key enzymes promoting gluconeogenesis. Additionally, HDACs are also involved in the gluconeogenesis process regulated by signal transducers and STAT3 [33]. The deacetylation of STAT3 by HDACs promotes transcription of STAT3 hepatic glycogenase in the hepatocytes of obese and diabetic patients. Accordingly, HDACs play an essential role in regulating hepatic glucose production.

## 3. Glucagon-Mediated Lipid Homeostasis in the Liver during the Fasting State

Lipids represent a necessary source of energy, particularly for the purposes of long-term storage. Lipids also protect the internal organs with skin, bones, and muscles, prevent the body temperature from spreading, and help the absorption of fat-soluble vitamins in food. Lipid metabolism is regulated by genetics, neurohumoral fluids, hormones, enzymes, and organs such as the liver. When these factors are abnormal, it brings about lipid metabolism disorders and pathophysiological changes in related organs such as hyperlipoproteinemia, lipid storage disease and its clinical syndrome, obesity, ketoacidosis, fatty liver, and neonatal scleredema [92]. As the central organ of lipid metabolism, the liver responds to nutrient and hormonal signals by regulating fatty acid oxidation and lipogenesis. It can synthesize lipoproteins, which is beneficial to lipid transport, fatty acid oxidation, and ketone body formation [93]. In the fasting state, the sugar supply is insufficient and glucagon secretion is increased, thus altering the acetylation state of the lipid metabolism enzyme, resulting in accelerated fat decomposition and increased ketone body formation (Figure 1B).

### 3.1. Glucagon-Induced Acetylation Enhances Fatty Acid Oxidation

When glycogen in the liver is depleted, the liver enhances oxidation of fatty acids to maintain the energy supply [94]. Fatty acid oxidation can not only provide a large amount of required energy, it is also the primary pathway for fatty acid decomposition and transformation in the body. The length fatty acid chains needed by the human body are different, and they are transformed by fatty acid oxidation. Therefore, chain fatty acids are turned into a suitable length for metabolism in the body [95]. Based on findings from many researchers, we summarize that glucagon can change the activity of fatty acid metabolism enzymes by acetylation in the liver, which is the most active organ for fatty acid oxidation, thereby enhancing fatty acid oxidation and increasing ketone body production. 

For example, glucagon raises the expression of sirtuin 3 (SIRT3) by enhancing the activity of PGC1-α in the fasting state [49,96,97]. SIRT3 is also an important member of the mammalian sirtuin family protein and plays a vital role in controlling metabolic activities [98,99]. Anderson et al. demonstrated that SIRT3 could regulate long-chain acyl-CoA dehydrogenase (LCAD) in the liver of mice through its deacetylation activity, increase LCAD levels, and enhance fatty acid oxidation. Thereby, it would reduce triglycerides and the accumulation of fatty acid oxidation intermediates affecting the metabolic syndrome [42]. LCAD is a crucial mitochondrial fatty acid oxidation enzyme. The defects of LCAD lead to fatty acid oxidation disorders and the accumulation of free fatty acids [100]. These results suggest that glucagon regulates LCAD by regulating SIRT3 and reduces the accumulation of free fatty acids (FFA). Additionally, Tong et al. found that accumulation of lipids was decreased by SIRT3-mediated motivation of the AMP-activated protein kinase (AMPK) in hepatocytes. The decrease in cellular energy storage results in reduction of the ATP/ADP ratio and an increase in the AMP/ATP ratio, while activation of AMPK promotes ATP synthesis, accordingly decreasing fatty acid synthesis and increasing fatty acid oxidation. This result suggests that glucagon may increase the oxidation of fatty acids through the activation of the SIRT3-AMPK signaling pathway [43]. And we can infer that glucagon-induced acetylation of energy-sensing factors by SIRT3 acts as a metabolic sensor in response to changes in cellular energy status.

The inhibitory effect of SIRT1 on gluconeogenesis might be an energy-saving means of the body, which would be reflected in lipid metabolism. With the extension of fasting time, glucose supply is insufficient and the body turns to lipid metabolism for energy supply. Under prolonged fasting conditions, the energy source is shifted from glucose metabolism to lipid metabolism in response to the insufficient supply of glucose [101]. Undoubtedly, this series of transformations is regulated by glucagon as the primary hormone that maintains energy balance during fasting. With the effect of glucagon, SIRT1 certainly regulates peroxisome proliferator-activated receptor-α (PPAR-α) to control hepatic lipid metabolism. PPAR-α is a nuclear receptor that is primarily located in organs with active lipid metabolisms, such as the liver [102]. The activation of PPAR-α promotes the utilization and catabolism of fatty acids by upregulating genes involved in fatty acid metabolism [103,104,105]. Therefore, as a lipid-sensing factor, activated PPAR-α modifies gene expression of proteins highly involved in the regulation of fatty acid metabolism, such as adipocyte fatty acid-binding protein (AFABP), fatty acid transporter (FATP), and lipoprotein lipase (LPL) [106,107,108,109]. Other research has found that the hepatocyte-specific deletion of SIRT1 undermined the activity of PPAR-α, which decreased fatty acid oxidation and led to development of hepatic steatosis and inflammation [110,111]. 

Furthermore, Ferdinand et al. revealed that glucagon-induced acetylation of Foxa2 was in control of lipid metabolism in response to fasting conditions [44]. The cofactors p300 and SIRT1, respectively, regulate Foxa2 acetylation and deacetylation at the Lys259 site [44]. During fasting, glucagon inhibits the activity of salt-inducible kinase 2 (SIK2) by activating adenylate cyclase (AC), thereby SIK2 decreases p300 activity [25]. Through this approach, glucagon improves the activity of p300 and further promotes the acetylation of Foxa2. Acetylation of Foxa2 increases the expression of genes involved in β-oxidation, such as carnitine palmitoyltransferase 1A (CPT1A) or medium-chain acyl-CoA dehydrogenase (MCAD) [45,46]. But SIRT1 can inactivate Foxa2 by deacetylation and thereby decrease the activity of Foxa2, which reflects a contradiction of SIRT1 in fatty acid metabolism [44,47]. The reason might be that SIRT1 improves the activity of PPAR-α by inhibiting Foxa2 because PPAR-α and Foxa2 competitively bind to the same promoter [112]. In addition, the concept that fasting increases SIRT1 activity has been oppugned [90,113]. Therefore, it deserves more attention to explore the different functions of SIRT1 and figure out its roles in the same metabolic process, which is helpful to clarify the metabolic mechanism. These results reflect that glucagon maintains the energy supply through different pathways under different nutritional conditions.

### 3.2. Glucagon-Induced Acetylation Inhibits Lipogenesis in the Liver

Lipogenesis in the liver is significant for the formation of very-low-density lipoprotein (VLDL) and the delivery of energy to other tissues, and this process is tightly regulated by hormones and nutritional status [114]. In the fasting state, because of an elevated glucagon concentration and activation of the intracellular cyclic adenosine monophosphate pathway, the acetylation status of lipid synthesis-related transcription factors is altered, resulting in low levels of de novo lipogenesis (DNL) [115]. In the fasting state, glucagon-mediated p300-CBP-associated factor (PCAF) acetylation and SIRT1 deacetylation pathways are involved in the acetylation of cAMP-responsive element-binding protein H (CREBH) to regulate hepatic lipogenesis [31]. As an energy-sensing factor for hepatic lipid metabolism, CREBH activates the expression of genes involved in the lipogenesis [116]. After glucagon stimulation, CREBH is acetylated by PCAF at the Lys294 site, which is necessary to interact with PPARα [31]. It has also been observed that the interaction of CREBH and PPARα synergistically increases fibroblast growth factor 21 (FGF21), leading to inhibition of lipogenesis in the fasting state [48]. Interestingly, after prolonged fasting, SIRT1 will in turn enhance the interaction of CREBH and PPARα [31]. This is compatible with the conclusion that SIRT1 plays distinct roles in different periods of fasting. In summary, this finding reveals that glucagon induces acetylation of CREBH to modulate lipid homeostasis in a time-dependent way.

In addition, glucagon has a significant regulatory effect on the activity of SREBP-1c through SIRT1-mediated acetylation modification. The transcription factor SREBP-1c works essentially on impacting transcription of hepatic genes such as glucokinase and fatty acid synthase. So, SREBP-1c positively regulates lipid synthesis by affecting the expression of the above genes [117,118]. In the fasting state, glucagon promotes the expression of SIRT1, and SIRT1 can respectively deacetylate SREBP-1c at Lys-289 and Lys-309. Deacetylation of SREBP-1c by SIRT1 decreases SREBP-1c activity and its association with lipogenic gene promoters [29]. In vivo experiments have also demonstrated that overexpression of SIRT1 reduces the stability of SREBP-1c, resulting in reduced lipid synthesis [119]. Besides, the function of SIRT1 in deacetylating the energy-sensing factor PGC-1α has been proved in fasting [84,120,121]. SIRT1-mediated deacetylation of PGC-1α increases its activity, which decreases lipid synthesis in response to glucagon [122,123,124]. These results indicate that glucagon precisely controls lipid synthesis by regulating the acetylation status of different energy-sensing factors.

## 4. Glucagon-Mediated Protein Homeostasis in the Liver

In health, the liver orchestrates the metabolism of proteins and amino acids. After proteins in food are broken down into amino acids (AAs) through the gastrointestinal tract, synthesis and metabolism of proteins in the body are re-executed mainly in the liver. The liver circulates urea to counter toxic ammonia produced in protein metabolism, thereby relieving the toxicity of ammonia. Thus, the urea cycle is the primary way for organisms to discharge nitrogen-containing metabolic waste [125,126,127]. During prolonged fasting, hepatic gluconeogenesis promotes carbon flux from AAs into central metabolism, when AAs become an important source of energy [127,128,129]. Under this condition, excessive ammonia is converted into urea to relieve ammonia poisoning [130,131]. As a hormone secreted mainly in the fasting state, glucagon maintains the metabolic balance of the body during CR by stimulating PGC1-α [120,132]. Research has shown that glucagon activates SIRT3 and sirtuin 5 (SIRT5) in the fasting state, which increases the activity of carbamoyl phosphate synthetase 1 (CPS1) and ornithine transcarbamylase (OTC) involved in ureagenesis. In this way, glucagon positively regulates ureagenesis by activating SIRT3 and SIRT5. SIRT3 and SIRT5 provide essential post-translational modification for a number of critical metabolic pathways [133,134,135]. Recent studies report that SIRT3 and SIRT5 promote ureagenesis in the fasting state [127,136]. During fasting, glucagon secretion stimulates the expression of PGC1-α in hepatocytes and alanine as a nitrogen source for urea production. PGC-1α enhances hepatic ureagenesis via promoting SIRT3 and SIRT5-mediated deacetylation of CPS1 and OTC [49,50]. This mechanism indicates that glucagon-induced acetylation of energy-sensing factors can also maintain metabolic homeostasis through ammonia detoxification during fasting, reflecting the diversity of glucagon biological functions.

## 5. Glucagon-Mediated Acetylation in Liver Disease

Hepatocytes are target cells of many hepatotoxic substances such as viruses, alcohol metabolites, and bile acids [137,138,139]. Therefore, the liver is vulnerable to attack by these hepatotoxic substances that contribute to liver metabolic syndrome [140]. It is well established that glucagon brings about elevation in plasma c-AMP and stimulates glycolipid metabolism in the liver. Undoubtedly, impairment of the liver will affect c-AMP biosynthesis, which is encouraged by glucagon, leading to compromised hepatic sensitivity to glucagon [141]. Multiple research studies have proven that metabolic disorders accompany an increase in plasma glucagon concentration [20,142]. We have introduced the critical role of glucagon-induced acetylation in liver metabolism. Further in-depth studies on its regulatory mechanisms and functions will contribute to improving hepatic metabolic diseases including NAFLD, hepatic fibrosis, and cancer.

### 5.1. Glucagon-Mediated Acetylation in Nonalcoholic Fatty Liver Disease (NAFLD) and Hepatic Fibrosis

Recently, NAFLD has been recognized to be one of the most common liver metabolic diseases in the world. NAFLD is thought to be a manifestation of metabolic syndrome in the liver, involving a series of disorders ranging from steatosis to steatohepatitis, with inflammation, liver damage, hepatocyte ballooning, glucose homeostasis, insulin resistance, and hepatic fibrosis [143,144,145]. Hepatic fibrosis is a pathological process characterized by the proliferation of extracellular matrix (ECM) after liver injury. Chronic hepatitis is accompanied by the progressive deposit of hepatic fibrosis, which may lead to cirrhosis. Patients with NAFLD and advanced hepatic fibrosis are at the highest risk for progressing to end-stage liver disease [146,147,148].

Recent investigations suggested that SIRT1, a critical metabolic regulator, and its enzymatic activity may be regulated by cellular energy, significantly improving disease progression in animal models of NAFLD [149,150,151,152]. It has been observed that the liver becomes insensitive to glucagon as a result of hepatic steatosis in NAFLD patients, which further promotes glucagon secretion [141]. However, in this state, the biological effect of glucagon is weakened, and the impact of glucagon on SIRT1 is also impaired, while SIRT1 can inhibit hepatic steatosis and inflammatory responses to hepatic metabolic disorders. Additionally, SIRT1 can reduce the level of oxygen consumption, which is correlative with NAFLD [153,154]. Conversely, hepatocyte-specific knockout of SIRT1 can cause significant hepatic steatosis and aggravate liver inflammatory responses. As a result, SIRT1 can deacetylate and modify STAT3, which will lead to STAT3 phosphorylation and lessen the activity of STAT3 [53,54]. STAT3 can regulate many target genes related to antiviral protection, hepatitis, and liver remodeling, and plays an essential role in liver fibrosis [54,155,156]. Additionally, glucagon can promote SIRT1 inhibition of liver inflammation, and inflammation is the most critical factor leading to the progression of liver fibrosis [157,158,159]. On the one hand, SIRT1 can down-regulate NF-κB activity and reduce inflammation [51,52]. On the other hand, SIRT1 can participate in fibrosis by regulating the transforming growth factor β (TGF-β) signaling pathway, which is very important in liver fibrosis [55,56]. These findings suggest that SIRT1 not only plays a crucial role in liver lipid metabolism-related diseases, such as NAFLD, but also plays a vital role in the development of liver fibrosis.

### 5.2. Glucagon-Mediated Acetylation in Tumorigenesis and Hepatocarcinogenesis 

Pathological changes in liver metabolism and physiological states will eventually lead to tumorigenesis and liver cancer. Hepatocellular carcinoma (HCC) is one of the end-stage liver diseases, and it has become the third leading cause of cancer mortality worldwide [160]. As a multi-factor, complex disease, the relationship between metabolic abnormalities and HCC has gradually been valued by researchers in recent years. Abnormal glycolipid metabolism is considered a potential risk factor for the development of HCC [161]. Cancer cells preferably generate lactate by the glycolysis pathway, even in aerobic conditions, to meet their demands of rapid growth and proliferation, known as aerobic glycolysis [162]. The main physiological functions of glucagon include the regulation of glycolipid metabolism and the inhibition of glycolysis, so there might be some association between tumorigenesis and HCC. High nuclear acetylation levels have also been observed in cancer cells for the increased activity of acetyltransferase [17,18]. Here, we review and discuss recent advances to elucidate how glucagon-induced acetylation of different energy-sensing factors has different effects on hepatocarcinogenesis and tumor growth. 

As mentioned above, glucagon can improve PCAF activity, and mounting evidence has revealed different effects of PCAF on HCC over the last few years. Overexpression of PCAF induces HCC cell apoptosis and autophagy, which is harmful for cancer cell proliferation [163]. In addition, PCAF induces acetylation of the K1323 site of PGK1, which in turn enhances the activity of deacetylase Sirtuin 7 (SIRT7) on K1323 and promotes cancer cell proliferation [57]. Pyruvate kinase M2 (PKM2) is acetylated at the K305 site by PCAF, which in turn leads to degradation of PKM2 [58,59]. PKM2 is expressed in different tissues and organs in which all large amounts of nucleic acids are synthesized, especially in tumor cells. Therefore, a high expression of PKM2 is accompanied by a variety of tumors, and this phenomenon is not caused by PKM2 splicing changes [164]. Ectopic expression of PCAF increases acetylation of PKM2, at K305 and decreases PKM2 activity. A decrease in the activity of PKM2 results in the accumulation of glycolytic intermediates upstream, such as fructose-1, 6-bisphosphate (FBP), and G6Pase [165,166]. So, the function of glycolysis is shifted from producing ATP to accumulating intermediate metabolites, providing raw materials for the synthesis of various biomacromolecules, thereby allowing tumor growth and cell proliferation. 

Glucagon-induced acetylation of PGC-1α and FOXO controls the expression of glycolytic genes. GCN5 acetylates PGC-1α and inhibits its transcriptional activity. The deacetylation of PGC-1α by SIRT1 in turn improves its activity, both of which modulate the balance of gluconeogenic and glycolytic genesis in hepatocytes [60,61]. FOXO transcription factors have been implicated in the upregulation of gluconeogenic genes and downregulation of glycolytic genes, playing a crucial role in tumor suppression [167]. Recent studies revealed the crucial role of FOXO acetylation in tumor suppression; activating FOXO will downregulate glycolytic genes. Glucagon-induced acetylation of FOXO by p300 inhibits its transcriptional and biological activities. In this regard, acetylation of FOXO could heighten glycolysis activity and promote cancer cell growth [62,63]. In summary, these findings may reveal that glucagon-induced acetylation of different energy-sensing factors has diverse effects on tumorigenesis and hepatocarcinogenesis, and also provides potential strategies for the treatment of liver cancer.

## 6. Concluding Discussion and Perspective 

In-depth studies on glucagon biology and pharmacology will help to further understand various modes in control of metabolism and provide potential therapeutic strategies for liver metabolic diseases. Researchers are aware that glucagon plays an unparalleled role in hepatic pathophysiology. Therefore, we reviewed glucagon-induced acetylation of energy factors in the control of hepatic metabolism, aiming to provide a treatment reference for liver metabolism diseases. 

Acetylation is a type of PTM in the nucleus, cytoplasm, mitochondria, and other organelles. Acetylation of energy-sensing factors is a significant regulator in hepatic metabolism. The number of nutrients in the environment and the changes in the types of nutrients can alter the direction of metabolism and the transformation between various metabolic pathways by affecting glucagon-induced acetylation of energy-sensing factors. Lysine acetylation modification of energy-sensing factors coordinates the interaction of various metabolic pathways well, and plays a fine role in the metabolic network of the organism. 

The discovery of glucagon-mediated acetylation of energy-sensing factors in control of liver metabolism opened new avenues of research into the biology and pharmacology of glucagon. Meanwhile, there are several important questions that need to be resolved, which are crucial for assessing whether glucagon-induced acetylation of different energy-sensing factors is a potential strategy for treating liver metabolism diseases:

1. The mechanisms by which glucagon regulates metabolic disorders remain unclear and require more relevant research.
2. It is still poorly understood how acetylation dynamically regulates the metabolic state of different cells and tissues and interacts with specific signaling pathways in response to changes of external environment.
3. Further understanding of the function and regulation of acetyltransferase and deacetylase will help to show how acetylation integrates different metabolic fluxes within cells and coordinates the entire metabolic network to meet the metabolic needs of cells.
4. Metabolic-related diseases have strong individual differences. Physiological and pathological changes in the acetylation state of different energy-sensing factors and their importance in the development of liver metabolism diseases still needs to be studied in depth, which not only contributes to detection and diagnosis of diseases, but also provides ideas for the further development of tissue-specific and metabolic pathway-specific drugs.

## Figures and Tables

**Table 1:** Overview of the regulation of different targets related to hepatic metabolism via glucagon-induced acetylation in different physiological states.

Physiological/Pathological State	Enzyme	Acetylation/Deacetylation	Targets	Effect	Metabolic Response	Reference
Fasting state	P300	Acetylation	CRTC2	Stimulatory	Gluconeogenesis↑	[25,34]
			FOXO1	Stimulatory	Gluconeogenesis↑	[26,35]
Fasting state	Ets-1	Acetylation	FOXO1	Inhibitory	Gluconeogenesis↓	[36]
Fasting state	SIRT6 and GCN5	Acetylation and Deacetylation	PGC1-α	Inhibitory	Gluconeogenesis↓	[37,38,39]
Fasting state	/	Acetylation	GP	Inhibitory	Gluconeogenesis↓	[40]
Prolonged fasting state	SIRT1	Deacetylation	CRTC2	Inhibitory	Gluconeogenesis↓	[25]
			FOXO1	Stimulatory	Gluconeogenesis↑	[25]
Fasting state	class IIa HDACs	Deacetylation	FOXOs	Stimulatory	Gluconeogenesis↑	[41]
Fasting state	SIRT3	Deacetylation	LCAD	Stimulatory	FFA oxidation↑ FFA accumulation↓	[42]
			/	Stimulatory	FFA oxidation↑FFA synthesis↓	[43]
Fasting state	SIRT1	Deacetylation	PPAR-α	Stimulatory	FFA oxidation↑	[44,45]
			Foxa2	Inhibitory	FFA oxidation↑	[46,47]
Fasting state	PCAF	Acetylation	CREBH	Stimulatory	FFA synthesis↓	[31,48]
Prolonged fasting state	SIRT1	Deacetylation	CREBH	Stimulatory	FFA synthesis↓	[31]
Fasting state	SIRT1	Deacetylation	SREBP-1c	Inhibitory	FFA synthesis↓	[29]
Fasting state	SIRT3 and SIRT5	Deacetylation	CPS1 and OTC	Stimulatory	Ureagenesis↑	[49,50]
NAFLD	SIRT1	Deacetylation	NF-κB	Inhibitory	Inflammation↓	[51,52]
Hepatic fibrosis	SIRT1	Deacetylation	STAT3	Inhibitory	Inflammation↓	[53,54]
Hepatic fibrosis	SIRT1	Deacetylation	TGF-β	Inhibitory	Inflammation↓	[55,56]
Liver cancer	PCAF	Acetylation	PGK1	Stimulatory	Glycolysis↑ Cancer cell proliferation and tumorigenesis↑	[57]
Liver cancer	PCAF	Acetylation	PKM2	Inhibitory	Tumor growth and cell proliferation↑	[58,59]
Liver cancer	GCN5	Acetylation	PGC-1α	Inhibitory	Glycolysis↑	[60]
Liver cancer	SIRT1	Deacetylation	PGC-1α	Stimulatory	Glycolysis↓	[61]
Liver cancer	P300	Acetylation	FOXO1	Inhibitory	Glycolysis↑ Cancer cell growth↑	[62,63]

## Abbreviations

AA	Amino acids
AC	Adenylate cyclase
AFABP	Adipocyte fatty acid binding protein
AMPK	AMP-activated protein kinase
cAMP	Cyclic AMP
CMA	Chaperone-mediated autophagy
COP1	Constitutively photomorphogenic 1
CPS1	Carbamoyl phosphate synthetase 1
CPT1A	Carnitine palmitoyltransferase 1A
CR	Caloric restriction
CREB	cAMP response-element-binding protein
CREBH	cAMP responsive element-binding protein H
CRTC2	CREB regulated transcription coactivator 2
DNL	De novo lipogenesis
ECM	Extracellular matrix
FATP	Fatty acid transporter
FBP	Fructose-1, 6-bisphosphate
FFA	Free fatty acids
FGF21	Fibroblast growth factor 21
FOXO1	Forkhead box O1
G6Pase	Glucose-6-phosphatase
GCN5	General control nonrepressed protein 5
GP	Glycogen phosphorylase
HCC	Hepatocellular carcinoma
HDACs	Histone deacetylases
HGP	Hepatic glucose production
HNF-4α	Hepatocyte nuclear factor 4α
KATs	Lysine acetyltransferases
LCAD	Long-chain acyl-CoA dehydrogenase
LPL	Lipoprotein lipase
MCAD	Medium-chain acyl-CoA dehydrogenase
MEK/ERK	Mitogen-activated protein kinase kinase-extracellular signal-regulated kinase
NAFLD	Nonalcoholic fatty liver disease
NF-κB	Nuclear factor kappa B
OTC	Ornithine transcarbamylase
PCAF	P300-CBP associated factor
PEPCK1	Phosphoenolpyruvate carboxykinase
PGC-1α	Peroxisome proliferator-activated receptor gamma coactivator 1α
PKA	Protein kinase A
PKM2	Pyruvate kinase M2
PPAR	Peroxisome proliferator-activated receptor
PPAR-α	Peroxisome proliferators-activated receptor-α
PTMs	Post-translational modifications
SIK2	Salt-inducible kinase 2
SIRT1	Sirtuin 1
SIRT3	Sirtuin 3
SIRT5	Sirtuin 5
SIRT6	Sirtuin 6
SIRT7	Sirtuin 7
SREBP-1c	Sterol regulatory-element-binding protein-1c
STAT3	Signal transducer and activator of transcription-3
T2DM	Type 2 diabetes
TGF-β	Transforming growth factor β
VLDL	Very-low-density lipoprotein
